# Acute tubulointerstitial nephritis in children– a retrospective case series in a UK tertiary paediatric centre

**DOI:** 10.1186/s12882-020-1681-7

**Published:** 2020-01-14

**Authors:** S. Roy, T. Awogbemi, R. C. L. Holt

**Affiliations:** 1 Paediatric Registrar, Department of Paediatric Nephrology, Alder Hey Children’s NHS Foundation Trust, Eaton Road, Liverpool, L12 2AP England; 2 Consultant Paediatrician, Department of General Paediatrics, Alder Hey Children’s NHS Foundation Trust, Eaton Road, Liverpool, L12 2AP England; 3 Consultant Paediatric Nephrologist, Department of Paediatric Nephrology, Alder Hey Children’s NHS Foundation Trust, Eaton Road, Liverpool, L12 2AP England

**Keywords:** Acute interstitial nephritis, Uveitis, Acute kidney injury, Paediatric

## Abstract

**Background:**

Acute tubulointerstitial nephritis (AIN) is an uncommon cause of acute kidney injury in children, accounting for less than 10% of cases. There is limited information regarding the range of underlying diagnoses and how these may differ geographically.

We undertook a retrospective case note review of consecutive cases of biopsy-proven AIN, presenting to a single UK tertiary paediatric centre, to describe the range of AIN in our caseload, define key characteristics and response to treatment, with the aim of informing paediatric nephrology practice.

**Methods:**

Cases were identified retrospectively from departmental records. Data extracted included demographics, presenting clinical and biochemical features, renal biopsy histology, treatment and follow-up.

**Results:**

Ten cases were identified over 8 years (2007–2014). Age range 6–16 years. Male:Female ratio 1:9. Final diagnoses included 6 tubulointerstitial nephritis and uveitis syndrome (TINU), 2 idiopathic, 1 sarcoidosis, 1 child with Streptococcal disease. Of the TINU cases, timing of eye symptoms varied in relation to AIN presentation. Cases had a varied investigative work-up.

Median presenting plasma creatinine was 303 μmol/l (range 152–932 μmol/l). Renal function improved spontaneously in 1 idiopathic case and improved with antimicrobial treatment in a child with Streptococcal disease.

Eight cases received immunosuppressive treatment with intravenous methylprednisolone (approximately 10 mg/kg for 3–5 days) and / or oral prednisolone (1–2 mg/kg initially, reducing over 7–28 days).

At 1 month, median creatinine had fallen to 91 μmol/l (range 41–120 μmol/l) with median eGFR 61 ml/min/1.73m^2^ (range 51-103 ml/min/1.73m^2^). At last follow-up (median 18.5 months, range 2–70 months), median creatinine was 71 μmol/l (range 47–90 μmol/l) with median eGFR 80 ml/min/1.73m^2^, range 63 to 101 ml/min/1.73m^2^).

Two patients received antihypertensives at diagnosis and 1 further patient at 1 month follow-up. Eight patients received electrolyte supplementation. Median time to discontinuing electrolyte supplementation was 3.5 months (range 1–12 months).

**Conclusion:**

To our knowledge, this is the only contemporary UK case series of biopsy-proven AIN in children. Our population has a high proportion of TINU. Treatment was accompanied by improvement of renal function, however 7/10 patients had an eGFR < 90 ml/min/1.73m^2^ at last follow-up. We suggest a standardised investigative work-up and recommend long-term follow-up.

## Background

Acute tubulointerstitial nephritis (AIN) is a relatively uncommon cause of acute kidney injury in children, accounting for less than 10% of all cases [1, 2]. AIN is an inflammatory condition affecting the renal interstitium, characterised by an infiltrate of T-lymphocytes, monocytes and eosinophils [3]; renal biopsy histology typically shows inflammation and damage of the tubulointerstitial structures with normal glomeruli and vessels [3]. Known causes include hypersensitivity reactions to medications (such as beta-lactam antibiotics, proton-pump inhibitors and non-steroidal anti-inflammatory drugs), infection-mediated AIN and autoimmune disorders [4]. Tubulointerstitial nephritis and uveitis syndrome (TINU) is a specific entity, which is thought to have an autoimmune basis, although its pathogenesis is not fully understood [4].

Previous publications have shown geographical variation in the range of underlying diagnoses, which may be related to international differences in prevalence of relevant infections and use of medications [5–8]. Unfortunately, there is a paucity of information available on AIN among children in the UK and limited resources to guide management. Our case series describes the presenting features, range of underlying causes and response to treatment for cases of biopsy-proven AIN, with the aim of informing contemporary practice in paediatric nephrology in similar settings.

## Methods

We performed a retrospective case note review of consecutive cases of biopsy-proven AIN presenting to a single UK tertiary paediatric centre, identified from our histopathology database. Data was extracted from both paper and electronic medical records and our electronic records database. Data included demographics, presenting clinical and biochemical features, renal biopsy histology, treatment and follow-up. Diagnosis of acute tubulointestitial nephritis was based upon classical histological appearances of immune cell infiltration [9]. All renal biopsies were performed and reviewed in the same centre. All of the patients were admitted to our centre within 24 h of initial presentation. Estimated glomerular filtration rate (eGFR) was calculated from height and plasma creatinine data using the Schwartz formula [10]. The constant of 40 is used in our centre.

## Results

Ten cases were identified over 8 years (2007–2014) with an age range 6–16 years and male:female ratio 1:9. Co-morbidities included cystic fibrosis (*n* = 1), asthma (*n* = 1) and chronic fatigue syndrome (*n* = 1). The duration of symptoms prior to presentation ranged from less than a week to several months (less than 1 month in 7/10 cases). Reported symptoms included polydipsia, nausea, vomiting, abdominal pain, reduced appetite, malaise, lethargy, joint pains, rash, eye pain and headaches. There was no documented history of oliguria/anuria. Three patients presented with a history of fever. 7/10 cases presented with vomiting and 1 patient was documented to have features of dehydration at presentation. See Table 1 for biochemical features at presentation.

**Table 1:** Biochemical features at presentation

Value	Median	Range
Plasma Creatinine	303	152–932 μmol/l
eGFR	19	7–31 ml/min/1.73m^2^
Potassium	3.5	2.8–4.1 mmol/l
Phosphate	1.1	0.59–2.04 mmol/l
Bicarbonate	16.5	14–20 mmol/l
Magnesium	0.92	0.73–1.27 mmol/l
C-reactive protein	24.5	< 4-233 mg/l
Erythrocyte sedimentation rate (ESR)^a^	99	27-142 mm/hr
Urinary retinol binding protein (RBP)^b^	20	2-90 mg/l
Urinary Albumin: Creatinine Ratio	22.1	7.7–387.8 mg/mmol

^a^ESR was performed in 8/10 patients

^b^RBP was quantified in 4/10 patients

At presentation, 8/10 patients had glycosuria, 4/10 patients had microscopic haematuria and 3/10 patients had leukocyturia, but microscopy revealed only one patient had urinary eosinophilia. Urine culture was negative in all patients. Virology testing for potential causes of AIN was performed in 5/10 patients and yielded negative results. Renal ultrasound was normal in 8/10 cases, with two cases showing increased echogenicity. Renal sizes in relation to height varied between the 50th and > 95th centiles [11].

Final clinical and histological diagnoses included 6 cases of TINU, 2 idiopathic AIN, 1 sarcoidosis and 1 AIN related to group A Streptococcal disease (See Table 2).

**Table 2:** Patient characteristics

Patient Number	Height (cm)	Weight (kg)	Lowest eGFR^a^ ml/min/1.73m^2^	Diagnosis	Timing of Eye Symptoms	Latest Follow up interval (months)	Latest eGFR^a^ ml/min/1.73m^2^	Electrolyte supplementation
1	158	36	31	Sarcoidosis		56	81	Potassium Acid Phosphate Magnesium Glycerophosphate Sodium Chloride Sodium Bicarbonate
2	162	39	22	TINU^b^	Post nephritis	2	66	
3	160.5	62.4	21	TINU^b^	Post nephritis	70	101	Potassium Chloride Potassium Bicarbonate Phosphate Sandoz
4	151.2	41.8	8	Idiopathic		39	63	
5	125.2	24.7	30	TINU^b^	Pre nephritis	26	91	Sodium Bicarbonate Phosphate Sandoz
6	167.5	75	7	TINU^b^	Concurrent	8	76	Sodium Bicarbonate Magnesium Glycerophosphate
7	156	57.55	18	TINU^b^	Pre nephritis	15	75	Potassium Chloride Sodium Bicarbonate Phosphate Sandoz Magnesium Glycerophosphate
8	115.3	23	25	Streptococcal		9	90	Magnesium Glycerophosphate
9	160.5	44.5	17	Idiopathic		22	89	Sodium Bicarbonate Phosphate Sandoz
10	158.6	49.85	7	TINU^b^	Concurrent	9	79	Sodium bicarbonate Phosphate Sandoz

^a^estimated glomerular filtration rate

^b^tubulointerstitial nephritis with uveitis

Among the TINU cases, the onset of eye symptoms was prior to AIN presentation in 2 cases, simultaneous in 2 cases and after AIN presentation in 2 cases. It is notable that one patient had been treated for uveitis 2 months prior to developing nephritis; in contrast, one patient had a negative ophthalmology screen on day 9 of admission and went on to develop uveitis at 4 month follow-up.

9/10 cases did not require renal replacement therapy. Our child with Streptococcal disease had an AKI Grade 3 with a rapidly rising urea up to 31.7 mmol/L within 53 h of presentation and, therefore, was treated by haemofiltration for approximately 24 h and thereafter continued to improve with targeted antimicrobial treatment. Renal function improved spontaneously in 1 idiopathic case.

The remaining 8 cases received immunosuppressive treatment with intravenous methylprednisolone, median 10 mg/kg (range 7-14 mg/kg) for 3–5 days and / or oral prednisolone (1-2 mg/kg initial dose and generally reducing over 7–28 days). There was no clear relationship between eGFR and whether the patients received IV or oral steroids and this decision was at the discretion of the treating clinicians. Among those patients who received IV methylprednisolone, 2 patients had a rapid rise in plasma creatinine within the first 24 h of presentation, which may have influenced the choice of treatment. Two patients received antihypertensive medication at diagnosis and a further 1 patient received antihypertensive medication 1 month after diagnosis. Eight patients received electrolyte supplementation (see Table 2).

**Fig. 1:**
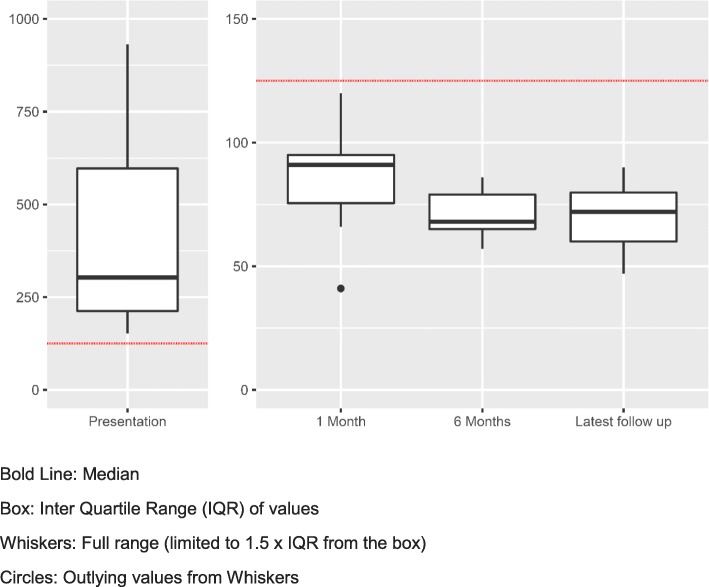
Box Plot to show plasma creatinine (μmol/l) at presentation through to latest follow up

At 1 month after presentation, median creatinine had fallen to 91 μmol/l (range 41–120 μmol/l) with median eGFR 61 ml/min/1.73m^2^ (range 51–103 ml/min/1.73m^2^). (See Fig. 1). Median time to discontinuing electrolyte supplementation was 3.5 months (range 1–12 months).

At last follow-up (median 18.5 months, range 2–70 months), median creatinine was 71 μmol/l (range 47–90 μmol/l) with median eGFR 80 ml/min/1.73m^2^ (range 63 to 101 ml/min/1.73m^2^). 7/10 patients had an eGFR of < 90 ml/min/1.73m^2^ at last follow-up (See Fig. 1)). None have experienced a recurrence of AIN.

## Discussion

We have summarised the findings of 10 consecutive patients with biopsy-proven AIN presenting to a single tertiary paediatric centre over 8 years. As far as we are aware, this is the only contemporary case series of AIN affecting UK children.

Patients typically presented with non-specific symptoms and severe renal impairment (estimated GFR around 30 ml/min/1.73m^2^ or lower). Only 20% were hypertensive at presentation. The clinical features of preserved urine output volume, plasma electrolyte abnormalities (metabolic acidosis with hypokalaemia and hypophosphataemia) and glycosuria reflected disturbances of renal tubular function due to tubulointerstitial inflammation. Albuminuria was relatively modest in most cases, consistent with impaired proximal tubular re-uptake, rather than the heavier albuminuria associated with primary glomerular disorders.

In the absence of features of chronic kidney disease, this constellation of clinical and biochemical features can be regarded as suggestive of AIN, but a renal biopsy is required for definitive confirmation. Although the presence of urinary eosinophils has previously been claimed to be a useful aid to diagnosis of AIN [3, 4], this was not supported by the findings in our case series. Short stature, established renal osteodystrophy or the demonstration of abnormally small kidneys on ultrasound scan should prompt evaluation for causes of chronic kidney disease that may present with a similar pattern of biochemical findings, such as nephropathic cystinosis and juvenile nephronophthisis [4].

Our case series has a high proportion of TINU cases, similar to that described by Jahnukainen et al. elsewhere in northern Europe [6], but differing from other case series published internationally [7, 8]. The high proportion of TINU cases underlines the importance of ophthalmological assessment during initial investigation and follow-up of children with AIN. The variable onset of uveitis is in keeping with the observations of the prospective study in Finland by Saarela et al. [7].

Other immunological causes of AIN include sarcoidosis, systemic lupus erythematosus, Sjogren’s syndrome and inflammatory bowel disease [3, 4, 12]. While acute interstitial nephritis in inflammatory bowel disease (IBD) may be associated with use of salicylate-derived medications, cases have also been recognised in IBD patients without such treatment [12]. Immunoglobulin G4 (IgG4)-associated immune complex multi-organ autoimmune disease is a rare disorder, which is characterised by elevated plasma IgG4 levels and can be associated with the development of AIN [12]. Our case series included a single case of sarcoidosis (Table 2), but none of these other disorders were represented. Collectively, these conditions appear to account for ≤10% of cases of AIN among children in the UK.

Historically, group A Streptococcal disease was regarded as a leading cause of AIN in autopsy series from North America [13]. Although this complication of Streptococcal infection is now less common in developed countries, case series from other parts of the world continue to include a high proportion of infection-related cases with geographical variation, dependent upon the regional prevalence of relevant pathogens [4, 5]. Infection can be complicated by AIN via either direct or indirect mechanisms. Direct renal invasion by micro-organisms may lead to local infection and inflammation, whereas the indirect mechanism involves a reactive inflammation with no evidence of renal infection [4].

The 2 idiopathic cases in this series had no relevant known medication exposures and were classified as idiopathic after an investigative work-up, which was not standardised and was dependent upon the clinical context and clinician.

Considering the underlying causes identified in our case series and the locally relevant infections, we recommend an investigative work-up for those children with suspected acute tubulointerstitial nephritis without an obvious medication-related aetiology (see Table 3). Where there is evidence of immunoglobulin deposition along tubular basement membranes on renal biopsy, consideration should be given to anti-tubular basement membrane antibody-mediated disease with appropriate further investigations [12].

**Table 3:** Recommended investigations for children with suspected acute interstitial nephritis, without a clear medication-related aetiology

Urine	
Urinalysis	
Microscopy, Culture and Sensitivity	
Albumin:Creatinine Ratio	
Retinol Binding Protein +/− urinary β2 microglobulin	
Blood	
Creatinine & Electrolytes (including Potassium/Bicarbonate/Magnesium/Calcium/ Phosphate)	
Full Blood Count	
Blood Culture	
EBV/CMV/Adenovirus PCR	
Mycoplasma Serology	
Toxoplasma Serology	
Anti-Streptolysin O Titre (ASOT) and Anti-DNase B Titre	
Complement C3 & C4	
Immunoglobulins A, G & M; IgG subclasses	
ANA/dsDNA/ANCA	
Angiotensin Converting Enzyme	
Imaging	
Renal Tract Ultrasound	
Chest X-Ray	
Other	
Ophthalmological Assessment	
Renal Biopsy	

In selected cases, such as immunosuppressed patients or those with a relevant travel history, further investigations may be indicated to identify BK polyoma virus nephropathy, or infections with human immunodeficiency virus, Mycobacteria, Legionella, Leptospira, Leishmania, Yersinia, Salmonella typhi, Brucella, Corynebacterium, hantavirus, fungi and rickettsiae [3, 4].

If renal biopsy reveals unexpected features of a more chronic disease process, it is warranted to consider an extended range of differential diagnoses, including the group of genetically-determined conditions falling within the category of autosomal dominant tubulo-interstitial kidney disease [12].

There have been no randomised controlled trials of therapies for AIN in children, but corticosteroid treatment has been advocated as beneficial in speeding up recovery of renal function [14] and was prescribed for 80% of the patients in our case series, in view of severe renal impairment without evidence of spontaneous improvement in these cases. Excretory renal function improved relatively quickly, with the largest improvement in eGFR occurring in the first 4 weeks after presentation. The majority of patients required electrolyte supplementation to correct deficiencies of potassium, bicarbonate, phosphate and magnesium during the recovery phase. Renal tubular function is difficult to systematically quantify from retrospective clinical data, but prolonged need for electrolyte supplementation suggests that recovery of renal tubular function was slower.

In TINU, uveitis generally requires additional treatment using topical corticosteroid preparations under the supervision of ophthalmology colleagues. While the interstitial nephritis component of TINU typically responds promptly to systemic corticosteroid treatment, the uveitis may be more refractory and require prolonged treatment, possibly involving additional immunosuppressive medications [12].

It has been reported that the prognosis for renal recovery is good [8]. However, 70% of our patients had an eGFR < 90 ml/min/1.73m^2^ at last follow-up, possibly placing these children at risk of hypertension or progression of chronic kidney disease in later life. Monitoring of blood pressure and renal function should continue long-term. We suggest follow up at 6–12 month intervals according to the individual patient characteristics and local arrangements.

Our case series is inevitably limited by its retrospective nature. Missing data at baseline included blood pressure in two patients and urine albumin: creatinine ratio in one patient. Nonetheless, it serves to highlight the range of diagnoses associated with AIN among children in the UK, along with the salient clinical features, the association with uveitis and importance of monitoring renal function in the long term.

## Conclusion

To our knowledge, we have described the only contemporary case series of biopsy-proven AIN in UK children. Our series of AIN has a high proportion of TINU cases. Appropriate treatment was accompanied by improvement of renal function. However, 7/10 patients had an eGFR of < 90 ml/min/1.73m^2^ at their last follow-up. Long-term follow-up is advised. We have recommended a standardised investigative approach with the aim of informing recognition, diagnosis and treatment of children presenting with AIN in similar settings.

## Data Availability

All data generated or analysed during this study are included in this published article.

## Abbreviations

AIN: Acute tubulointerstitial nephritis
eGFR: Estimated glomerular filtration rate
TINU: Tubulointerstitial nephritis with uveitis
